# Lupus Nephritis in an Adolescent Girl With Hyper-Immunoglobulin E

**DOI:** 10.7759/cureus.34332

**Published:** 2023-01-29

**Authors:** Ghadeer G Alahmadi, Sherif M El-Desoky, Reem A Al Zahrani, Jameela A Kari

**Affiliations:** 1 Pediatrics, King Abdulaziz University Faculty of Medicine, Jeddah, SAU; 2 Pediatrics Nephrology, King Abdulaziz University Faculty of Medicine, Jeddah, SAU; 3 Pathology, King Abdulaziz University Faculty of Medicine, Jeddah, SAU

**Keywords:** immune complexes, immune dysregulation, systemic lupus erythematosus, lupus nephritis, hyper ige syndrome

## Abstract

We report the case of an adolescent girl with frequent hospital admissions for severe eczematous skin rashes with recurrent epistaxis and chest infections. Investigations revealed persistent severely elevated serum total immunoglobulin E (IgE) levels but normal levels of other immunoglobulins, suggesting hyper-IgE syndrome. The first skin biopsy revealed superficial dermatophytic dermatitis (tinea corpora). Another biopsy performed after six months revealed a prominent basement membrane with dermal mucin, suggesting an underlying autoimmune disease. Her condition was complicated by proteinuria, hematuria, hypertension, and edema. A kidney biopsy revealed class IV lupus nephritis, according to the International Society of Nephrology/Renal Pathology Society (ISN/RPS). Based on the American College of Rheumatology/European League Against Rheumatism (ACR/EULAR) criteria, she was diagnosed with systemic lupus erythematosus (SLE). She was first administered with intravenous pulse methylprednisolone (600 mg/m^2^) for three consecutive days, followed by oral prednisolone (40 mg/m^2^) daily, mycophenolate mofetil tablets (600 mg/m^2^/dose) twice daily, hydroxychloroquine (200 mg) once daily, and three classes of antihypertensive medications. She maintained normal renal functions with no lupus morbidity for 24 months, then rapidly progressed to end-stage kidney disease, and was then started on three to four sessions of regular hemodialysis per week.

Hyper-IgE is known to be a marker of immune dysregulation as it facilitates the generation of immune complexes (ICs) that mediate lupus nephritis and juvenile SLE.

Regardless of the different factors that are impacting the production of IgE, the present case illustrated that juvenile patients with SLE may have increased IgE levels, indicating that higher IgE levels might have a role in lupus pathogenesis and prognosis. The mechanisms regarding the increased levels of IgE in subjects with lupus need further investigation. Further studies are thus required to assess the incidence, prognosis, and possible new specific management for hyper-IgE in juvenile SLE.

## Introduction

Systemic lupus erythematosus (SLE) is an autoimmune disease caused by autoantibodies and circulating immune complexes (ICs), in which the immune system attacks its own tissues. This causes inflammation and tissue damage in the affected organs [1].

The hallmark of SLE is excessive production of antibodies to nuclear antigens (ANA), double-stranded DNA (dsDNA), ribonuclear proteins (RNP), Smith antigen (Sm), ribonuclear proteins (RNP), Ro (SSA), La (SSB), and some phospholipids; these antibodies accumulate over time. SLE pathogenesis is closely associated with the presence of dsDNA-specific immunoglobulin G (IgG) antibodies [2].

However, kidney biopsies reveal kidney deposits comprising dsDNA IgG antibodies in numerous patients with SLE who develop glomerular nephritis [3]. Furthermore, other isotypes of autoantibodies may be associated with lupus activity; thus, kidney pathology remains a topic of considerable interest.

IgE is associated with immunity against various parasitic infections and is involved in mediating type I hypersensitivity in mast cells and basophils. It is considered the rarest immunoglobulin in blood, with serum levels of approximately 150 ng/mL in a healthy human body.

The presence of IgE autoantibodies has been observed in some autoimmune diseases along with the prevalence of autoreactive IgE in SLE; however, its importance in the disease activity and pathologies remains unclear [4]. Previous studies have demonstrated that SLE pathophysiology occurs because of loss of tolerance toward various nuclear antigens such as dsDNA, Sm, and RNP, resulting in activated pathogenic autoantibodies, particularly from the IgG isotype, along with IgE. Circulating ICs are formed and deposited in target organs initiating tissue-damaging inflammation, which plays a major role in SLE pathogenesis. Thus, autoreactive IgE antibodies and circulating ICs containing IgE have been detected in the serum of patients with SLE [5].

## Case presentation

Case history

We report the case of an adolescent 11-year-old Burmese girl who presented to King Abdulaziz University Hospital with a diffuse, progressive, itching skin rash all over her body and scalp for seven months. The rash initially manifested on her back and abdomen, then progressed to the face and head, and subsequently to her entire body, including the palms and soles. It progressed to crusting and oozing clear fluid, associated with a three-week history of fever and bilateral eye swelling. She had a history of moderate unilateral epistaxis, which only required nasal packing in the emergency room; she had no other history of bleeding, petechia, or bruising. She was not on any specific therapy.

She was delivered at full term through spontaneous vaginal delivery, and her perinatal history was irrelevant, with incomplete vaccinations because of social reasons. She was admitted to a hospital when she was four months old for bronchiolitis with secondary bacterial pneumonia. She was living with her non-consanguineous parents and her seven healthy siblings. The family belonged to a low socioeconomic status. There was no family history of rheumatological, immunological, renal, or any chronic illnesses.

Physical examination

An initial assessment revealed a low-grade fever (<38.5°C) but otherwise stable vital signs. She was not in distress and was well-hydrated with a normal capillary refill. Her weight was 23 kg (fifth centile), and her height was 125 cm (25th centile for age and sex). She was conscious, alert, and oriented. She was pale with erythematous maculopapular, non-blanchable, crusted, and pruritic skin rashes all over her body with scales including the palms and soles without any exudate. Her right eye was swollen and congested, but with normal eye movement.

The remaining clinical examinations yielded unremarkable findings. Table 1 summarizes the initial and accumulative laboratory workup. She was hospitalized to investigate the cause of her skin rash and fever. Sepsis was ruled out; however, she was empirically administered intravenous ceftriaxone. Considering her severe skin rash, high lactate dehydrogenase and ferritin, as well as low fibrinogen, she was advised bone marrow trephine and skin biopsy; both tests have ruled out hemophagocytic lymphohistiocytosis (HLH), Langerhans cell histiocytosis (LCH), and macrophage activation syndrome secondary to autoimmune versus reaction.

**Table 1:** Summary of initial and accumulative subsequent workup CBC: Complete blood count; Hb: Hemoglobin; WBC: White blood cells; ENA: Extractable nuclear protein; C3: Complement 3; C4: Complement 4; ESR: Erythrocyte sedimentation rate; CRP: C-reactive protein; ACA: Anti-cardiolipin antibodies; PT: Prothrombin time; INR: International ratio; APTT: Activated partial thromboplastin time; LDH: Lactate dehydrogenase; HBs-Ab: Hepatitis B surface antibodies; HCV-ab: Hepatitis C virus antibodies; HIV: Human immune deficiency virus; CMV: Cytomegalovirus; EBV: Epstein-Barr virus; ALP: Alkaline phosphatase; AST: Aspartate aminotransferase; ALT: Alanine transaminase; GGT: Gamma-glutamyl transferase.

	Initial workup	At 2 w	At 4 w	At 3 m	At 6 m	At 12 m	At 18 m	At 24 m	At 30 m
CBC									
Hb [N: 12–15 g/dL]	8.8	7.7	12.7	11.1	6.7	9.4	8.2	5.9	8.3
Platelet [N: 150–400 K/µL]	170	58	158	235	294	339	468	458	196
WBC [N: 4–13 K/µL]	4.42	3.01	6.68	6.07	8.14	10.74	15.94	23.8	6.2
Neutrophil [N: 1–8.5 K/µL]	1.2	3.51	1.40	1.62	3.24	3.2	11.85	18.7	4.3
Basophil [N: 0.01–0.08 K/µL]	0.01	0.07	0.04	0.02	0.02	0.01	0.02	0.03	0.00
Eosinophil [N: 0.2–0.8 K/µL]	0.00	0.01	0.00	1.00	0.12	0.04	0.00	0.02	0.00
Monocyte [N: 0.4–1 K/µL]	0.09	0.99	1.02	0.62	0.99	0.97	1.40	1.69	0.34
Lymphocyte [N: 1.5–6.8 K/µL]	0.60	2.63	1.43	2.99	2.07	3.81	2.14	2.27	1.49
Immune profile									
ANA	1:680	-	-	-	1:1280	1:1280	1:1280	1:1280	1:1280
RNP ENA	-	-	-	-	-	+ve	-	-	+ve
SM ENA	-	-	-	-	-	+ve	-	-	+ve
Anti-dsDNA [N: 0–200 IU/mL]	119.8	-	-	-	114.1	1,104	338	956	367
ACA-G [N: 0–20 CU]	-	-	-	-	-	12.7	<2.6	-	-
ACA-M [N: 0–20 CU]	-	-	-	-	-	4.4	2.6	-	-
Lupus anticoagulant 1[N: 0–45]	-	-	-	-	32.9	33.4	30.4	-	-
C3 [N: 0.75–1.65 g/L]	0.96	1.06	-	-	0.21	0.48	0.33	0.42	0.52
C4 [N: 0.2–0.6 g/L]	0.35	0.34	-	-	0.04	0.06	0.08	0.08	0.21
ESR [N: 0–20 mm/hr]	-	-	-	103	102	104	78	-	92
CRP [N: 0–3 mg/L]	4.2	18	10.9	13	16.7	39.6	12.3	58	3.2
Direct Coomb's test IgG	+ve	-	-	+ve	+ve	+ve	+ve	-	-
IgE [N: 0–390 KU/L]	>5,000	-	-	-	>5,000	>5,000	-	-	-
IgG [N: 5.4–16.1 g/L]	15.5	-	-	22.6	17.9	5.38	5.7	-	-
IgA [N: 0.5–2.5 g/L]	2.48	-	-	2.64	2.08	1.99	2.06	-	-
IgM [N: 0.5–1.8 g/L]	0.75	-	-	0.95	0.73	1.15	0.99	-	-
Coagulation profile									
PT [N: 4.9–12.5 sec]	11.9	-	-	11.4	11.6	11.6	9.4	10	-
INR [N: 0.85–1.3 ratio]	1.06	0.89	0.86	1.01	1.0	1.03	0.78	0.8	-
APTT [N: 25.1–36.5 sec]	62.3	41.4	33.5	-	31.6	33.1	24.9	24.3	-
D-Dimer [N: 0–0.5 mg/dL]	4.4	4.4	1.6	-	-	-	-	14.6	-
Fibrinogen [N: 200–393 mg/dL]	339	181	497	-	-	-	-	405	-
Ferritin [N: 13–150 ng/mL]	4,155	3,533	40,000	1,034	247	336	748	614	745
LDH [N: 100–240 U/L]	976	880	-	-	-	-	-	330	382
Serology									
HbsAg	-ve	-	-	-		-	-	-ve	-ve
HBs-Ab [N: 0–10 mU/mL]	2.50	-	-	-		-	-	2.89	2.04
HCV-ab	-ve	-	-	-		-	-	-ve	-ve
HIV 1&2	-ve	-	-	-		-	-	-ve	-ve
Virology									
CMV-IgG	+ve	-	-	-	-	-	-	-	-
CMV-IgM	-ve	-	-	-	-	-	-	-	-
EBV Ab-IgG	+ve	-	-	-	-	-	-	-	-
EBV Ab-IgM	-ve	-	-	-	-	-	-	-	-
Parvovirus B19 Ab-IgM	-ve	-	-	-	-	-	-	-	-
Parvovirus B19 Ab-IgG	-ve	-	-	-	-	-	-	-	-
Liver function test									
Total protein [N: 64–82 g/L]	73	-	73	68	79	68	46	68	76
Albumin [N: 40.2–47.9 g/L]	29	31.8	32.6	27.4	36.1	23.9	26	38	40
ALP [N: 141–460 U/L]	53	122	92	84	63	63	66	130	178
AST [N: 15–37 U/L]	91	64	35	30	15	18	16	81	47
ALT [N: 12–78 U/L]	44	88	27	13	12	16	11	90	11
GTT [N: 5–85 U/L]	39	36	20	26	18	31	16	165	17
Kidney function and urine tests									
Creatinine [N: 49–90 umol/L]	46	27	24	-	34.9	137	48	268	106
Urea [N: 3.2–8.2 mmol/L]	2.8	2.7	3	2.2	1.5	3.1	3.3	6.1	8.9
Urine creatinine [N: 5300–22100 µmol/L]	7550	-	-	-	-	2,445	1,789	22,494	-
Proteinuria (random) [N: 0–20 mg/L]	10	-	-	-	-	1,271	666	3,800	-
Albuminuria/Creatinine ratio [N: 0–30 mg/g]	20	-	-	-	-	4,595	3,292	1,493	-

Furthermore, the patient exhibited a positive immune profile for SLE, even though the characteristic clinical manifestations were absent. The ANA pattern was speckled with a moderate positive titer (1:640), 119.8 dsDNA, positive direct Coombs test (direct agglutination test) as IgG +1, weakly positive perinuclear antineutrophil cytoplasmic antibodies (p-ANCA), and negative c-ANCA. Thus, the immunology team was consulted. A primary immune deficiency was ruled out considering the time of presentation; however, partial innate immune defects and hyper-IgE syndrome were considered in her differential diagnoses. A genetic study using whole exome sequencing was performed, and the result was negative for possible variants responsible for hyper-IgE syndrome. Therefore, IgE and other immunoglobulin assays were performed. A definitive diagnosis of SLE was difficult during that time owing to the lack of clinical manifestations and negative lupus-specific serology.

Hospital course

**Figure 1:**
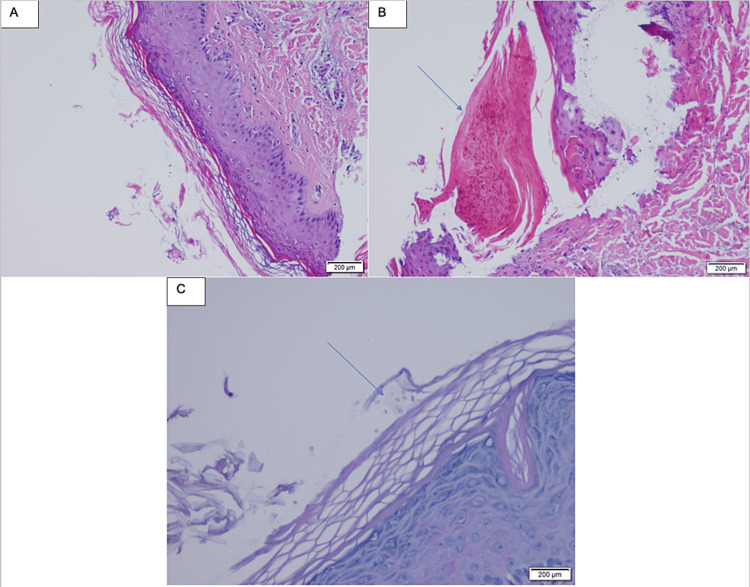
First skin biopsy: (A) orthokeratosis and parakeratosis, (B) parakeratosis (arrow), and (C) rare fungal spores (PAS-D stain) PAS-D: Periodic acid-Schiff with diastase.

During her hospital stay, the skin rash was initially crusty and covered the entire body and scalp. Skin biopsy (Figure 1) from the left thigh was consistent with tinea corpora; therefore, she was administered betamethasone and ketoconazole. Furthermore, two weeks into her hospital stay, she developed acute chest pain with tachycardia and desaturation. An electrocardiogram revealed a T-wave inversion. Chest radiography revealed bilateral interstitial lung infiltration as well as right pleural effusion with right upper lobe collapse. The patient had to be shifted to the intensive care unit for severe respiratory failure, which entailed three days of non-invasive ventilation.

**Figure 2:**
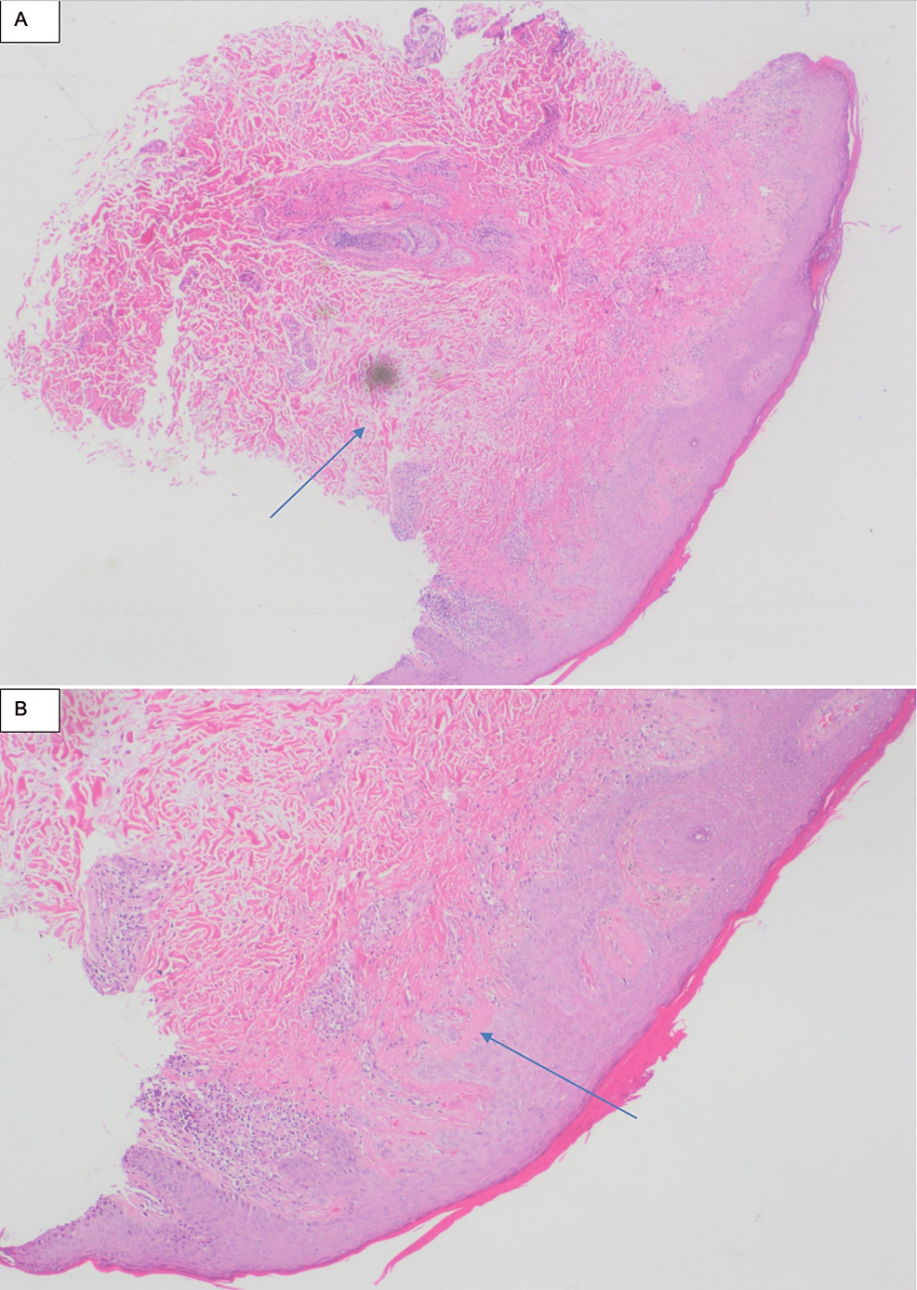
Second skin biopsy showed (A) increased dermal mucin (as indicated by the arrow tip) and (B) a thick basement membrane shown at higher magnification (indicated by the arrow)

Her hemoglobin levels dropped, with pancytopenia (Table 1). Skeletal and skull radiography and bone marrow biopsy with flow cytometry yielded unremarkable results with no evidence of HLH or LCH. She required fresh frozen plasma, packed red blood cell and platelet transfusions, vitamin K, and tranexamic acid. In the pediatric intensive care unit (PICU), she developed generalized tonic-clonic convulsions; however, her computed tomography and magnetic resonance imaging yielded normal results. Afterward, she was treated with levetiracetam (30 mg/kg/day). Skin biopsy repeated after six months (Figure 2) revealed a prominent basement membrane with dermal mucin, suggesting an underlying autoimmune disease. Moreover, her serum IgE levels were extremely high (>5,000 KU/L [0-390]) on repeated tests. The genetic study using whole exome sequencing looking for possible variants responsible for the hyper-IgE syndrome like signal transducer and activator of transcription 3 (STAT3) gain-of-function (GOF) germline mutations sent to CENTOGENE GmbH, Germany, yielded negative results.

The patient required multiple admissions over the following year due to recurrent infections at 6, 12, and 18 months from her initial presentation; subsequent investigations at six months revealed strong positive ANA (1:1280) but negative anti-dsDNA. However, at 12 months, her investigations revealed persistently positive ANA, low C3 and C4, positive anti-Smith antibodies, and strong positive anti-dsDNA (Table 1). Subsequently, clinical manifestations of SLE such as painless oral ulceration on the hard palate and annular alopecia were observed. Therefore, she was diagnosed with active SLE and prescribed mycophenolate mofetil (600 mg/m^2^/dose twice daily), oral prednisolone (1 mg/kg/day), hydroxychloroquine (200 mg), omeprazole (20 mg), and enalapril (20 mg) once per day to treat hypertension associated with significant proteinuria.

**Figure 3:**
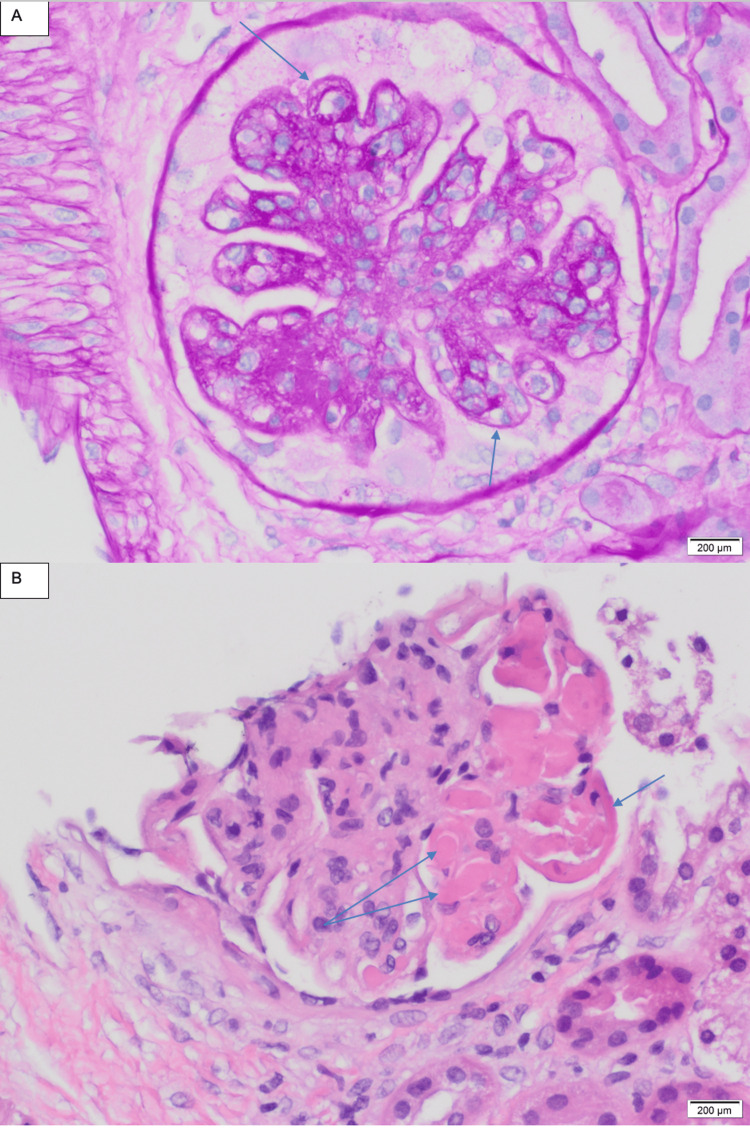
Light microscopic findings from a kidney biopsy: (A) a glomerulus with mesangial proliferation, endocapillary hypercellularity (short arrow), and segmental narrow double contour (long arrow), PAS stain; (B) a glomerulus with prominent subendothelial immune deposits in the form of wire-loop lesion (one short arrow) and hyaline thrombi (two long arrows), H&E stain PAS: Periodic acid-Schiff; H&E: Hematoxylin and eosin.

**Figure 4:**
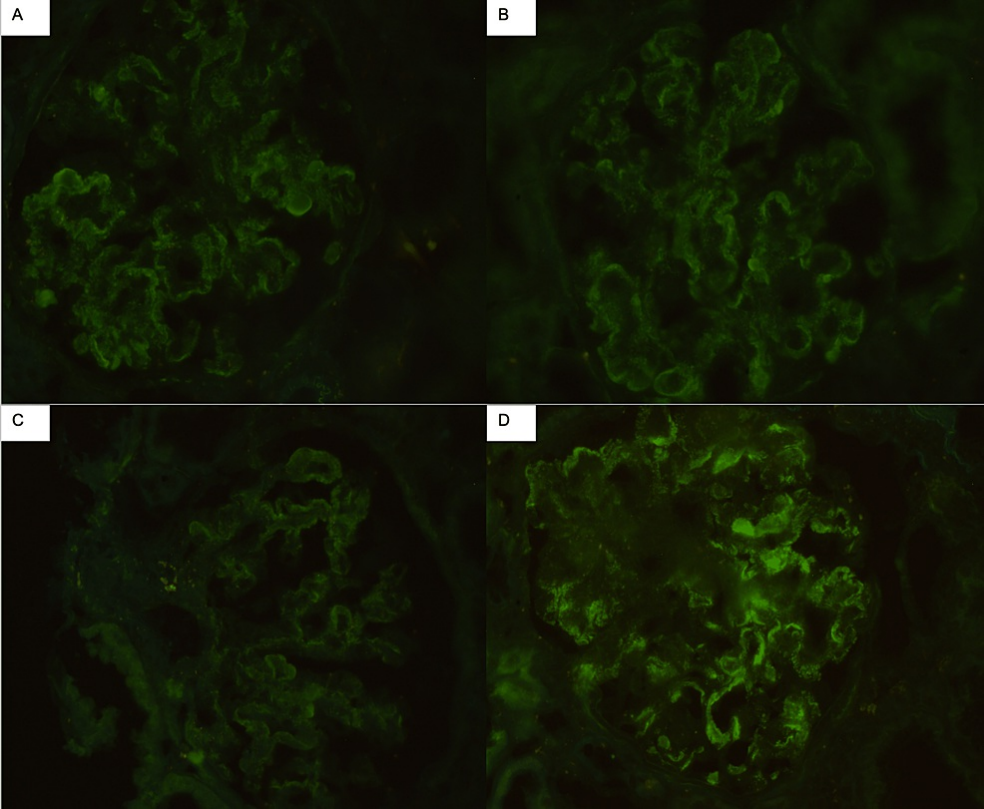
Kidney biopsy, direct immunofluorescence staining, granular mesangial and capillary wall staining. (A) IgG of 1+ intensity. (B) IgM of 1+ intensity. (C) IgA of 1+ intensity. (D) C1q of 2+ intensity. C3, kappa, and lambda light chain stains were of the same distribution and intensity of IgG (not shown).

Subsequently, she exhibited generalized edema, anemia, heavy proteinuria, hematuria, and hypertension (Table 1). She was then diagnosed with active lupus nephritis. Renal biopsy revealed diffuse membranoproliferative glomerulonephritis on light microscopy (Figure 3). Direct immunofluorescence staining demonstrated considerable mesangial and capillary wall staining for IgG, IgA, and IgM as well as kappa and lambda light chains (full house pattern) in the glomerular mesangium and capillary walls (Figure 4). Staining for C1q was more prominent than C3 and the other previously mentioned immunoglobulins and light chains. Therefore, a diagnosis of class IV (SN/RPS) diffuse lupus nephritis was rendered. She was then administered IV pulse methylprednisolone (600 mg/m^2^ surface area daily) for three days, followed by oral prednisolone (40 mg/m^2^ surface area) daily, along with similar doses of mycophenolate mofetil and hydroxychloroquine, and three blood pressure lowering agents (an angiotensin-converting enzyme inhibitor, calcium channel blocker, and non-selective beta receptors blocking agent) at the maximum allowed doses.

She retained normal renal function for 24 months after the first presentation, but her kidney functions acutely deteriorated with generalized anasarca 12 months from the diagnosis of lupus nephritis. She was admitted through the emergency room to the PICU with a clinical picture of cardiorespiratory failure, hypertension, oxygen desaturation on room air, and acute rise in renal function profile with oliguric renal failure. X-ray chest and heart showed a picture of acute pulmonary edema with cardiomegaly. She was managed with positive pressure ventilation and urgent daily hemodialysis sessions. Re-induction of immune suppression for control lupus activity was done according to our hospital protocol with daily intravenous pulse methylprednisolone 500 mg/m^2^ for three consecutive days, followed by intravenous rituximab 375 mg/m^2^ together with cyclophosphamide 100 mg for two doses one week apart. She was maintained on full-dose oral daily prednisolone 60 mg/m^2^ with a maintenance dose of oral mycophenolate mofetil 600 mg/m^2^/dose twice daily. Unfortunately, her kidney function did not improve after four weeks of intensive management. The suggestion to repeat kidney biopsy was considered, but her lack of response to re-induction, immune suppression, and the original histopathological diagnosis of diffuse proliferative lupus nephritis deferred our decision to repeat the biopsy. She continued to receive regular hemodialysis three to four sessions per week, together with maintenance immunosuppressive treatment for persistently active SLE.

## Discussion

In this case, the diagnosis of SLE was delayed for one year based on the initial clinical picture and persistently high serum IgE levels, which indicated hyper-IgE syndrome. In addition to the initial negative lupus-specific immunological tests (anti-dsDNA), which were strongly positive after 12 months of initial presentation, the case was complicated simultaneously by active lupus nephritis. Kidney biopsy revealed class IV lupus nephritis based on ISN/RPS. The activity index was 6/12, and the chronicity index was 0/12 based on the modified National Institute of Health (NIH) scoring system [1]. Then, her condition progressed to end-stage kidney disease over 12 months of SLE diagnosis.

Limited studies have illustrated an association between juvenile SLE and elevated serum IgE concentrations, despite the existence of atopic symptoms or parasitic disease. Owing to the complexity of SLE pathogenesis, the reasons for these findings and their associations with the disease risk and characteristics remain unresolved [2].

Previous studies have shown that elevated IgE production secondary to disturbance of immune tolerance could lead to polyclonal activation of B lymphocytes and aberrant Th2 responses along with the production of autoantibodies. Additionally, cells with the IgE isotype will be associated with the overproduction of interleukins (IL)-4, IL-5, IL-10, and IgE, which is primarily found in patients with parasitic infections and atopic diseases [3,4].

Furthermore, IgE overproduction has been reported in patients with partial T cell immunodeficiency and autoimmune diseases, which are usually associated with overactivity of the adaptive immune system [4,5]. These observations indicate that an imbalance between the immunogenic and tolerogenic signals in T cells causes an increase in IgE levels, which may be considered a biomarker of immune dysregulation [6,7].

Previous reports have demonstrated that elevated total IgE levels may be associated with a history of allergic and connective tissue disease owing to the release of vasoactive mediators from the basophils and mast cells. IgE is associated with an increase in vasopermeability, which may be significant in the deposition of circulating ICs in glomerulonephritis. However, some studies have assumed that the pathogenesis of connective tissue disease is not caused by an increased level of IgE [8,9]. However, other studies have demonstrated that SLE pathophysiology involves a loss of tolerance toward nuclear antigens such as dsDNA, Sm, and RNP among others. This results in the auto-reactivation of T and B cells by autoantigens leading to the development of autoantibody-secreting plasmablasts [10]. The resulting pathogenic autoantibodies are predominantly of the IgG isotype as well as IgE, leading to high serum levels of IgE, which will form circulating ICs; these circulating ICs deposit in target organs and initiate a tissue-damaging inflammation, which plays a major role in SLE pathogenesis [10].

IgE deposition was not observed in the immunofluorescence study of the kidney biopsy from the current patient. However, IgE serum levels are reportedly increased in lupus patients with kidney involvement, with the detection of IgE immune complex deposition in kidney biopsies [11]. Moreover, high IgE levels have been observed in adult patients with SLE without kidney involvement, suggesting that IgE may contribute to SLE pathogenesis itself, and not only nephritis [12].

Studies in adult patients with SLE have identified an important correlation between disease activities as they have IgE levels at least two folds higher than those in patients with inactive disease. Moreover, IgE kidney deposits in patients with lupus patients correlate with a poor prognosis. This observation conflicts with that of previous studies of patients with SLE, which showed similar levels of IgE levels in subjects with or without nephritis [13].

IgE levels inversely correlate with C4 levels, suggesting that the complement cascade is activated, and components are consumed. This was observed in the present case by the reduction of C4 level, which is a marker of disease activity, and the high IgE level, which is a purported marker of disease activity [14].

Several studies have suggested the term “auto-allergy” to indicate the autoimmune processes that are mediated by IgE autoantibodies [15]. There are two forms of IgE in SLE: autoreactive IgE and non-autoreactive IgE. The autoreactive IgE has been linked with high SLE activity and lupus nephritis and encourages the production of interferon (IFN) alpha by plasmacytoid dendritic cells (pDC) [6,16]. Activation of basophils by ICs with autoreactive IgE amplifies autoantibody production [17]. Non-autoreactive IgE is negatively associated with the lupus erythematosus activity index (SLEDAI) and prevents the release of IFN by pDC [18]. Studies have revealed that 30%-50% of SLE patients with autoreactive IgE are mainly directed against dsDNA, Ro/SSA, La/SSB, and Sm [2,19], and anti-dsDNA IgE was discovered in all lupus subtypes [16] and have been linked with the activity of SLE disease [20] and kidney involvement. Reducing the levels of autoreactive IgE and inhibiting their receptors or increasing non-autoreactive IgE are considered promising therapeutic objectives in the management of SLE [18].

Interestingly, high levels of serum IgE were noticed in children whose mothers have lupus disease, despite the existence of allergic disease in the mothers [20].

## Conclusions

Hyper-IgE is considered a marker of immune dysregulation and may be important in the generation of ICs that mediate juvenile SLE and lupus nephritis. Despite the various factors influencing IgE production, the present case demonstrated that juvenile patients with SLE may have higher IgE concentrations, suggesting that IgE overproduction could play a role in lupus pathogenesis.

The specific mechanisms underlying the elevation of IgE levels in children with lupus remain to be clarified, and further studies are needed in this regard. Juvenile lupus nephritis with hyper-IgE may carry a worse prognosis; therefore, early detection is strongly recommended. Further studies are needed to assess the incidence, prognosis, and possible new specific management for hyper-IgE in juvenile SLE and to understand the causality and associations of hyper-IgE in juvenile SLE patients.
